# Primary Biliary Cholangitis With Incomplete Response to Conventional Therapies But Had Complete Response to Baricitinib: A Case Report

**DOI:** 10.1155/crhe/9033401

**Published:** 2025-12-28

**Authors:** Gi Eun Kim, Mariam Imran, Sherif Mostafa, Yasser Kamel

**Affiliations:** ^1^ Department of Internal Medicine, Hamad General Hospital, Hamad Medical Corporation, Doha, Qatar, hamad.qa; ^2^ Department of Gastroenterology, Ambulatory Care Center, Hamad Medical Corporation, Doha, Qatar, hamad.qa

**Keywords:** alkaline phosphatase, baricitinib, case report, primary biliary cholangitis

## Abstract

Primary biliary cholangitis is a progressive disease with complications such as liver cirrhosis and hepatocellular carcinoma, and the treatment goal is to delay its progression. One of the markers for treatment response is alkaline phosphatase levels. Baricitinib has been used in one randomized controlled trial involving two patients to improve outcomes in unresponsive primary biliary cholangitis. We present a case of primary biliary cholangitis who had incomplete response to ursodeoxycholic acid, obeticholic acid, and fenofibrate but showed complete response to baricitinib in terms of sustained normalized alkaline phosphatase levels.

## 1. Introduction

Primary biliary cholangitis (PBC) is a chronic autoimmune condition characterized by lymphocytic destruction of the small bile ducts [1]. The serologic hallmark of the disease is antimitochondrial antibody (AMA), which is positive in 90%–95% of patients with PBC [2]. Most patients are asymptomatic at diagnosis but gradually develop symptoms over the next two decades. The most common and disabling symptoms is fatigue, closely followed by pruritus. Although the presence or absence of symptoms does not correlate with disease prognosis, symptoms can significantly affect a patient’s quality of life [2]. The rate of progression varies, but it usually progresses over decades to cirrhosis and decompensated liver failure requiring liver transplant, and it predisposes patients to other complications such as hepatocellular carcinoma, esophageal varices, and osteoporosis [1].

First‐line therapy for PBC is ursodeoxycholic acid (UDCA), which improves liver biochemistries and survival without liver transplant in long term [1]. Obeticholic acid (OCA) was approved by the Food and Drug Administration (FDA) for combination use with UDCA or as an alternative in patients with PBC with inadequate response to UDCA after 1 year of the treatment. However, recently, OCA has been voluntarily withdrawn from the US market in September 2025 due to risk of severe liver injury. Fibrates can be used as off‐label alternatives in those with inadequate response to UDCA [1].

Although UDCA has been shown to improve liver transplant–free survival and reduce mortality in patients with PBC, around 40% of patients with PBC have inadequate response to UDCA after 1 year [3]. For those with inadequate response to the available therapies, there are limited options and the need for an alternative therapy is high. Baricitinib has risen as a potential treatment for PBC unresponsive to first‐line therapy [4]. We present a case of PBC that was partially responsive to UDCA, OCA, and fibrates but showed complete response in terms of alkaline phosphatase (ALP) normalization on baricitinib.

## 2. Case Presentation

A 50‐year‐old woman initially presented in 2018 due to cholestatic rise in liver enzymes associated with itchiness and fatigue for 1 year. She was diagnosed with PBC based on positive AMAs and ultrasound of the abdomen showing no biliary dilatation. She was started on UDCA 1500 mg daily with partial improvement in her symptoms and partial response of her liver enzyme levels (Table 1). Liver biopsy was performed, which was consistent with PBC with possible autoimmune hepatitis overlap. She was placed on a trial of prednisolone 40 mg daily for 2 weeks, with no improvement in liver enzymes; thus, it was withdrawn. Due to persistently elevated liver enzymes after 2 years of UDCA, she was started on fenofibrate 200 mg daily with improvement of her itchiness but no improvement in her liver enzymes. Three months after starting fenofibrate, she was placed on OCA, which was gradually titrated up to 10 mg daily, with no improvement in her liver enzymes.

**Table 1:** Trend of liver enzymes.

	25/9/2017 (prior to diagnosis of PBC)	28/8/2019 (2 years into UDCA treatment)	03/01/2024 (prior to starting baricitinib)	20/03/2024 (3 weeks into starting baricitinib)	08/05/2024 (2 months into starting baricitinib)	18/08/2024 (5 months into starting baricitinib)	Reference
Total bilirubin	12.3	9.6	10	9	5	8	0–21 μmol/L
ALP	807	276	253	114	195	94	35–104 U/L
ALT	133	42.5	44	42	31	29	0–33 U/L
AST	99	36	43	39	31	37	0–32 U/L

Her other comorbidities include obesity with body mass index (BMI) of 39.6 kg/m^2^, prediabetes with HbA1c 5.7%, chronic kidney disease Stage 3 with unknown etiology as kidney biopsy was refused by the patient, osteoporosis, and alopecia areata for more than 20 years. She developed alopecia totalis, for which she was started on baricitinib 2 mg daily on 7/3/2024, because of relative contraindication for other systemic treatments due to her comorbidities. This was done with a close follow‐up with the gastroenterology team with repeat liver enzymes every 4 weeks. Her ALP levels showed a significant decrease of 50% within 3 weeks of starting baricitinib, a slight increase at 2 months, and then a continued downward trend to normalization at 5 months of starting baricitinib (Table 1). ALT and AST levels have also decreased, while the total bilirubin level was within the normal range from the start (Table 1). US elastography performed after 3 months of baricitinib showed an average stiffness of 9.4 kPa, compared to 12.5 kPa in US elastography performed 1 month prior to starting baricitinib in Egypt. Her symptoms were well controlled during this period. She was kept on baricitinib as she showed excellent response in terms of alopecia totalis, and up till now, there were no adverse effects of baricitinib 8 months into treatment.

## 3. Discussion

Baricitinib is a Janus kinase (JAK) inhibitor which is thought to inhibit several cytokines associated with PBC including Type 1 interferon, Interleukin 12, and Interleukin 23 [4]. Many key cytokines associated with different autoimmune diseases such as rheumatoid arthritis, psoriasis, and IBD use JAK pathway; thus, many autoimmune diseases are theoretically amenable to JAK inhibitors as treatment [5]. Currently, baricitinib is approved for use in moderate to severe rheumatoid arthritis [6]. There is evidence that there is clinical improvement with baricitinib in patients with rheumatoid arthritis [7, 8], psoriasis [9], and systemic lupus erythematosus [10]. In one randomized controlled trial of two patients, baricitinib was used in one patient with PBC who had inadequate response to UDCA, which showed 30% decrease in ALP level, 7‐point improvement in itch NRS, and improvement in marker of inflammation (CRP) and liver fibrosis score over a 12‐week period [4]. The baricitinib‐treated patient in the study was a 65‐year‐old female who was diagnosed with PBC 14 years ago, with early cirrhosis on elastography, with hypertension and dyslipidemia, and was treated previously with only UDCA. The ALP level prior to treatment initiation was similar to that of our patient, ranging from 224 to 240 U/L. There was a slight increase in ALP levels to 253 U/L at around 4 weeks of the treatment, after which there was a consistent decrease to 157 U/L. Within 4 weeks of stopping baricitinib, ALP levels increased back to pretreatment levels [4]. In our case, there was an initial decrease in ALP levels followed by a slight increase at 2 months, after which there was a consistent downward trend. Although there was a numerical decrease in liver stiffness in liver elastography, as the two US elastography were done in different centers, it might be difficult to conclude that the fibrosis score also improved.

Several studies have shown that bilirubin and ALP levels are good predictors of long‐term outcomes [11, 12]. One meta‐analysis showed that patients with ALP levels ≤ 2 times the upper limit of normal (ULN) at 1 year had a 10‐year survival rate of 84% compared to 62% in patients with ALP levels > 2 times the ULN, with similar results of improved survival with normal bilirubin levels compared to high bilirubin levels [11]. In another cohort study, achieving normal ALP levels and bilirubin level of < 0.6 of ULN were associated with a lower risk of liver transplant or death [12]. Clinical guidelines also recommend lowering the ALP levels to at least two times the ULN [1].

Two peroxisome proliferator‐activated receptor (PPAR) agonists, elafibranor and seladelpar, have received accelerated approval from the FDA in 2024 [13]. However, these are not yet available in Qatar.

The strengths of our case report are the compelling temporal pattern between the start of baricitinib and the improvement in the ALP level within 3 weeks, as well as the sustained response observed while the patient is on the treatment. The trend of the ALP level increasing at 1‐2 months after the start of baricitinib also matches with the trend shown in the randomized control trial. The limitations of the case report are the lack of US elastography of the liver done in the same center to accurately assess for improvement in liver stiffness, and due to the prolonged nature of the disease, the lack of long‐term outcomes in the patient including possible relapse when baricitinib is stopped.

We present a case of PBC partially responsive to UDCA, fibrates, and OCA but showed complete and sustained response to baricitinib over 8 months that was started for another indication. Further randomized controlled trials testing this novel potential therapy for PBC unresponsive to conventional therapies should be carried out.

## Ethics Statement

Ethics committee approval was waived.

## Consent

Written informed consent was obtained from the patient for publication.

## Conflicts of Interest

The authors declare no conflicts of interest.

## Funding

There was no financial support for this case report.

## Data Availability

The data that support the findings of this study are available on request from the corresponding author. The data are not publicly available due to privacy or ethical restrictions.
